# Heart failure in Nigeria: protocol for a systematic review and meta-analysis

**DOI:** 10.4314/ahs.v23i2.61

**Published:** 2023-06

**Authors:** Okechukwu S Ogah, Oyewole A Kushimo, Adewole Adebiyi, Chuka T Onyema, Onubogu C Uchenna, Bosede E Adebayo, Oyinkansola T Agaja, Olanike A Orimolade, Amina Durodola, Isa A Makinde, Rufus O Akinyemi, Waheed A Adedeji, Ademola J Adekanmi, Joshua O Akinyemi, Babatunde Adedokun

**Affiliations:** 1 Cardiology Unit, Department of Medicine, University of Ibadan, Ibadan, Oyo State, Nigeria; 2 Cardiology Unit, Department of Medicine, University College Hospital Ibadan, Oyo State, Nigeria; 3 Institute of Cardiovascular Diseases, College of Medicine, University of Ibadan, Oyo State, Nigeria; 4 Cardiology Unit, Department of Medicine, Lagos University Teaching Hospital, Lagos, Nigeria; 5 Cardiology Unit, Department of Internal Medicine, Alex Ekwueme Federal University Teaching Hospital, Abakaliki, Ebonyi, Nigeria; 6 Paediatric Cardiology and Respiratory Unit, Department of Paediatrics, Rivers State University Teaching Hospital, Port Harcourt, Rivers State, Nigeria; 7 Paediatric Cardiology Unit, Department of Paediatrics, University College Hospital, Ibadan, Oyo State, Nigeria; 8 Cardiology Unit, Department of Internal Medicine, Federal Medical Center, Abeokuta, Ogun State, Nigeria; 9 Neuroscience and Ageing Research Unit, Institute of Advanced Medical Research and Training, College of Medicine, University of Ibadan, Nigeria; 10 Clinical Pharmacology Department, University College Hospital, Ibadan, Nigeria; 11 Pharmacology and Therapeutics Department, College of Medicine, University of Ibadan, Nigeria; 12 Department of Radiology, College of Medicine, University of Ibadan, Nigeria; 13 Department of Epidemiology and Medical Statistics, College of Medicine, University of Ibadan, Nigeria; 14 Center for Observational Research, Amgen, Thousand Oaks, California, United States

**Keywords:** Heart failure, cardiac failure, cardiac dysfunction, heart dysfunction, systematic review, meta-analysis, Nigeria

## Abstract

**Background:**

Heart failure is now a significant contributor to the burden of non-communicable diseases in developing countries like Nigeria which is experiencing epidemiologic and demographic transition. The epidemiology of heart failure in this country is poorly characterized. The aim of the review is to determine the prevalence of heart failure, the associated risk factors, the aetiology, management, and outcomes of the condition in the country.

**Methods:**

Relevant databases such as PubMed /Medline, EMBASE, Web of Science, Google Scholar, African Index Medicus, and African journal online would be searched for articles published in English from January 2000 to December 2021. The analysis will include observational studies conducted among Nigerian adults aged 12 years and above. Article selection shall be conducted by pairs of independent reviewers. Data extraction shall be done by 2 independent reviewers.

**Results:**

The primary outcome would be the pooled prevalence of heart failure while the secondary outcomes would be to identify the risk factors and management of heart failure in Nigeria.

**Conclusion:**

This will be the first systematic review and meta-analysis of heart failure epidemiology in Nigeria which will hopefully identify gaps for future research and guidance for policy interventions.

## Introduction

Heart failure (HF) has emerged as a major public health problem. 1,2 The syndrome is now an issue of global importance. 2 It has been established that the burden of HF doubles with each passing decade after the age of 40 years due to aging population and increasing burden of risk factors including hypertension, diabetes mellitus, ischaemic heart disease and, more recently obesity. 3

HF affects over 64.3 million people worldwide.^4^ It is a highly symptomatic syndrome that affects 1-3% of the population in high income countries especially in people above the age of 65 years.^5^, ^6^ Approximately 15 million (out of 900 million) Europeans and 5.8 million (out of 300 million) Americans are affected by HF. 7, 8 In the United States of America (USA) alone, up to 2.4 million hospital admissions can be attributed to HF as primary or secondary diagnoses. 9 It is the primary reason for 1215 million office visits and 6.5 million hospital-days each year.^9^ About 670,000 new cases occur in the USA yearly.^9^

Data from developed countries show that half of HF patients are hospitalized within 6-months of discharge 27 and 70% of these are due to worsening of previously diagnosed HF. 10 Jencks et al 11 showed that HF is the number one reason for readmission in the USA. HF readmission is associated with higher mortality compared to the index admission

HF is associated with shorter life expectancy, greater morbidity and impaired quality of life than most common diseases. About 30% of people die within 3 months of diagnosis of HF. Annual mortality is 10% thereafter. In high-income countries, the economic burden of HF is high because it is associated with frequent hospital admissions. 12, 13 Moreover, management of HF places significant financial burden on patients, their families/ care-givers and, as indicated, society as a whole. 12, 13 Over 39 million dollars is spent annually in the USA for the care of HF patients. In Europe, over 60% of economic cost of HF is related to hospital admissions. 13 This is because HF is associated with high rates of hospital admissions, readmissions and frequent clinic visits. 13 In response, strategies for the effective management of the disease in the outpatient setting have been developed and applied. This has led to improved survival in some countries. 7, 14, 15

Cardiovascular diseases are increasingly being recognized in Africa as a result of increased aging among the population as well as better strategies aimed at the control of communicable diseases and malnutrition.^16^ A rapid epidemiological transition has been observed in sub-Saharan Africa, with an increased prevalence of cardiovascular risk factors such as hypertension, diabetes mellitus, hyperlipidaemia and obesity.^16^ Recent data from sub-Saharan Africa on acute HF shows that the leading causes of HF are hypertension, rheumatic heart disease, cardiomyopathies and ischaemic heart disease. It affects mainly those of middle age (in the prime of their life) 16 Mortality at 6-months is about 17.8% 16 and twice this rate at 12 months. 17-19

In most developing countries, the burden of HF has not been well characterized as there are no reliable population-based methods for estimating the incidence, prevalence as well as prognosis of the condition. This is principally because of differences in the diagnostic criteria as well as the definition of the condition. A recent systematic review found that hypertensive heart disease, cardiomyopathies and rheumatic heart diseases were most common in sub-Saharan Africa, while ischemic heart disease was relatively uncommon. Additionally, the review revealed high readmission and mortality rates and a significant burden of the disease among young adults. 20

There is a lack of information on the epidemiology of HF in Nigeria (Africa's most populous country). We therefore plan to use a systematic approach to obtain all available information on the subject in the country, summarize the data and provide a guide for future research and policy direction.

## Objective

The aim of the systematic review is to determine the prevalence of HF, the associated risk factors, the aetiology, management and outcomes in Nigeria.

### Review questions

The specific review questions to be addressed are;

- What is the demographic profile of HF in Nigeria?
- What are the risk factors for HF in Nigeria?
- How is HF managed in Nigeria?
- What are the outcomes of HF in the country?

## Methods

A systematic review of published work on HF in the country shall be carried out. This shall be done using the guidelines for meta-analyses of observational studies 21 as well as the Preferred Reporting Items for Systematic review and Meta-analysis (PRISMA) guidelines. 22 This protocol is registered to PROSPERO (reg no: CRD42022315547)

### Information Sources/Study identification

A literature search shall be conducted of articles published (in English language, the official language in Nigeria,) between January 2000 and December 2021 in the major databases: PubMed /Medline, EMBASE, Google Scholar, Web of Science. African index medicus and African journal online.

We shall also review the reference lists of retrieved articles journals in order to obtain additional studies. Published abstracts shall be included if they contain information needed for the analysis.

### Search Strategy

A detailed search strategy shall be developed for the extraction of the articles. An initial limited search of PubMed shall be carried out in order to identify the relevant keywords contained in the title, abstract and subject descriptions. The initial search terms will be “heart failure” OR “Cardiac failure” and Nigeria. Thereafter, these search terms as well as their synonyms will then be used for extensive search in PubMed and other databases. In addition, a search shall be performed to answer other research questions using the terms “heart failure” and the risk factors/ outcomes of interest. Filters shall be used to narrow down to articles in English language published during the study period. A detailed search term following the PICO (Patient/Population/ Problem, Intervention/ Prognostic factor, Comparison, Outcome) format shall be employed. 23, 24 (Table 1 shows the planned search strategy)

**Table 1:** The Search strategy that will be employed in the review

SNO	Database	Search strategy
1	Medline/PubMed	Search: (“Nigeria”) AND (“cardiac failure” OR “heart failure” OR “acute heart failure” OR “chronic heart failure” OR “acute cardiac failure” OR “chronic cardiac failure” OR “cardiac decompensation” OR “heart decompensation” OR “cardiac dysfunction” OR “heart dysfunction” OR “left heart failure” OR “right heart failure” OR “systolic dysfunction” OR “diastolic dysfunction” OR “congestive cardiac failure” OR “congestive heart failure” OR “biventricular heart failure” OR “thyroid heart disease” OR “thyrotoxic heart failure” OR “hypertensive heart failure” OR “high output heart failure” OR “endomyocardial fibrosis” OR “peripartum cardiomyopathy” OR “cor pulmonale” OR “dilated cardiomyopathy” OR “restrictive cardiomyopathy” OR “myocardial infarction” AND (2000:2021[pdat]))
2	Embase	(‘heart failure’: ab,ti OR ‘cardiac failure’: ab,ti OR ‘heart dysfunction’:ab,ti OR ‘cardiac dysfunction’:ab,ti OR ‘congestive heart failure’:ab,ti OR ‘congestive cardiac failure’:ab,ti) AND nigeria AND [2000-2021]/py
3	Web of science	(((((((TI= (heart failure)) OR TI= (cardiac failure)) OR TI= (heart dysfunction)) OR TI= (cardiac dysfunction)) OR TI= (Congestive cardiac failure))) OR TI= (Congestive heart failure)) AND ALL= (Nigeria)
4	Google scholar	Heart failure” AND “Nigeria” “Cardiac failure” AND “Nigeria”
	African Index Medicus	“Heart failure” AND “Nigeria” “Cardiac failure” AND “Nigeria”
5	African Journal online	“Heart failure” AND “Nigeria” “Cardiac failure” AND “Nigeria”

### Eligibility Criteria

The analysis will include all observational studies (cross-sectional studies and cohort studies) conducted in the country during the study period.

### Inclusion Criteria

All full text articles from observational studies (cross-sectional, cohort, retrospective or prospective) that meet the search criteria and published in English language from Jan 1 2000 to December 31, 2021 will be included in the review.

### Exclusion Criteria

We shall exclude case reports, case series, editorials, Comments, expert opinions as well as letters to the editor due to lack of peer review. Qualitative studies shall also be excluded.

### Participants

Only studies conducted in adult Nigerians aged 12 years and above and in which at least 50 subjects were recruited shall be included. When there is a duplicate publication, the study with the larger sample shall be included.

### Measures of the results

We shall collect information on the first author, year of study (if available), year of publication, location and region of the study, sample size, study design, mean age, age range, proportion of females, method of diagnoses/diagnostic criteria, aetiology of HF, co-morbidities, management (drugs and procedures) and outcomes (length of hospital stay and mortality)

### Data collection process, article selection and data extraction

The selection and processing of the study shall be carried out using standard procedure. The selection process will be done in two stages. In the first stage, two pairs of reviewers shall independently select the articles by evaluating the titles and abstracts according to our pre- established criteria. The agreement between the choices made by these reviewers will be evaluated using kappa's statistic and any discrepancies will be assessed and a consensus reached for eligibility by a third reviewer who is the most senior cardiologist on the team. In the second stage, the full text of all the articles identified from stage 1 will be downloaded and two reviewers will similarly examine the articles for eligibility and any discrepant assessments settled as for stage 1.

For ineligible articles, the reason for rejection will be recorded and the frequency distribution of these reasons documented. All the relevant data shall be extracted into an excel spreadsheet. Discrepancies shall be resolved by consensus. Where necessary, the first authors of the articles shall be contacted for useful information.

### Evaluation of risks of bias

We shall evaluate for the risk of bias using the STROBE checklist. 25 This will be based on the information provided in the methodology of each article. We shall evaluate the selection of participants, control of confounding factors, measurement as well as reporting of the results as well as conflicts of interest.

Independent pairs of reviewers shall evaluate the quality of the methods used by the authors of the identified articles. Where there is discrepancy, this shall be resolved by consensus. The cohort type studies version of the ROBINS- I assessment shall be used. 26

### Data Abstraction and Collection

A standardized data abstraction form will be developed to extract relevant data from full text articles using the PRISMA checklist as a guide. Two independent reviewers will pilot test the abstraction form using five selected articles to assess validity and reliability. Piloting will assess the content and consistency of abstracted data. Following the pilot, a lead reviewer will review and abstract data from the articles. A second independent reviewer will repeat the extraction to verify the information extracted, and any discrepancies will be resolved by a third reviewer. Full copies of all the selected articles shall be used for data analysis. All the selected articles shall be organized with the Endnote reference manager. Extracted data shall be exported to the Rayyan web app for systematic reviews for adequate sorting. 27

Required information for the analysis shall be extracted into an excel sheet. Two reviewers shall independently perform this. Discrepancy shall be resolved by consensus with other members of the team.

### Statistical Analysis

Calculations of the standard error (SE) and effect size (ES) of the prevalence estimates shall be done using metaprop one. Unadjusted prevalence estimates of HF shall be calculated based on the information of crude numerators and denominators provided by individual studies. To keep the effect of studies with extremely small or extremely large prevalence estimates on the overall estimate to a minimum, the variance of the study-specific prevalence shall be stabilized with the Freeman-Tukey double arc-sine transformation before pooling the data with the random-effects meta-analysis model.

When data were available by gender group, we will compare proportions between males and females. Funnel plot and Egger test will be used to assess publication bias and will be considered significant if the p value of Egger was <0.05.

We shall use forest plots to present data on individual studies as percentages with exact binomial 95% confidence intervals (CIs). The studies shall be pooled by geopolitical zone of the country using the random effects method of DerSimonian and Laird. 17 Heterogeneity chi-square (χ^2^) test and Tau^2^ statistic (τ^2^) shall be used to assess the heterogeneity and the between-study variances in which p-values less than 0.05 will be considered as heterogeneous. Individual means shall be weighted by study size in pooled analyses, and the pooled mean and the range of means shall be presented. I^2^ statistic will be carried out to test for variance in ES attributable to heterogeneity and differences between the geopolitical zones. Tables will be used to present data on individual studies as prevalence and exact binomial 95% confidence intervals (CIs). Meta-regression will be used to ascertain the predicting causes of heart failure from the list of aetiologies reported by the different included studies against increases in years of study on heart failure in Nigeria.

Random effects meta-regression shall also be performed to determine if the year of study is an explanation for the between-study heterogeneity in aetiology of HF, management, and mortality. Corresponding bubble plots shall be drawn, with the size of each bubble inversely proportional to the estimated variance in the respective study.

Patients presenting acutely to hospitals may differ in many respects from those that are seen in clinics for chronic management. When pooling the data, we shall therefore indicate the setting of each study in all forest plots. Studies shall be sub-analysed separately (for acute, chronic and both in- and out-patient populations), and an overall pooled estimate of HF prevalence also calculated. The quality and risk of bias of all included studies will be assessed using the risk of bias assessment tool developed by Hoy et al. ^36^. This tool shall be adapted for the different topics on heart failure that will be covered in the review e.g prevalence, aetiology, treatment and prognosis of heart failure.

All statistical analysis shall be carried out using STATA Version 14 (Stata Corp. College Station, Texas, USA).

## Discussion

Prevalence of CVD risk factors such as hypertension, diabetes and obesity are presently on the rise in Nigeria. 28,29 A recent large prospective registry study also suggests the rising incidence of acute coronary syndromes in this country. 30 These trends suggest the likely rise in the burden of heart failure. The epidemiology of heart failure is poorly characterized in Nigeria due to the absence of large population-based studies. To the best of our knowledge there is currently no systematic review or meta-analysis of the epidemiology and clinical outcomes of heart failure in Africa's most populous country. This review will help to describe and synthesize the current landscape of heart failure in Nigeria, while we await the conduct of population studies. This will hopefully identify gaps in knowledge for future research and also guide policy for unmet needs of heart failure subjects in this region.

## Strengths and limitations of the study

To the best of our knowledge, this will be the first systematic review and meta-analysis of heart failure studies in Nigeria. The pooled sample from different regions of the country will likely be a strength to this work.

A possible limitation of this study will be the inclusion of predominantly non-randomized hospital-based studies which will preclude the generalizability of our results to the general Nigerian population. Furthermore, some eligible studies may be missed by our search strategy because some work may not have been published in visible platforms. Nevertheless, the identification protocol will be made as inclusive as possible. To mitigate the risk of missing relevant articles, we will use broad headings and many databases. Hence, the comprehensive search and inclusion of available data will provide the most robust and contemporaneous overview of heart failure in our country.

Systematic reviews are potentially susceptible to publication bias. We shall also attempt to limit the potential for publication bias by conducting an extensive search for all relevant publications reporting prevalence or providing data from which prevalence can be calculated. Two independent reviewers will extract the data, and study authors will be contacted where age range is unclear.
